# Atorvastatin Combining with Probucol: A New Way to Reduce Serum Uric Acid Level during Perioperative Period of Interventional Procedure

**DOI:** 10.1155/2014/565367

**Published:** 2014-01-29

**Authors:** Hong Li, Ximing Li, Hongjun Ma, Yiran Wang, Naikuan Fu, Dongxia Jin, Hongliang Cong

**Affiliations:** ^1^Graduate School, Tianjin Medical University, Tianjin 300051, China; ^2^Department of Geriatrics, The First Hospital of Qinhuangdao, Qinhuangdao 066000, China; ^3^Department of Cardiology, Tianjin Chest Hospital, Tianjin Medical University, Xi'an Road No. 93, Heping District, Tianjin, 300051, China; ^4^Cardiology Department, Dagang Oil Field General Hospital, Tianjin Medical University, Tianjin 300051, China

## Abstract

Uric acid has ever been considered as one of contrast induced acute kidney injury's risk factors. Atorvastatin and probucol can both improve contrast induced acute kidney injury separately. This prospective study is to assess their effect on reducing serum uric acid level and contrast induced acute kidney injury during perioperative period of interventional procedure. On the basis of different doses of atorvastatin and probucol, 208 cases admitted for coronary angiography or percutaneous coronary intervention were randomly classified into standard combined group (S-C group), intensive combined group (I-C group), and intensive atorvastatin group (I-A group). Patients' blood urea nitrogen, serum creatinine, and serum uric acid were measured and estimated glomerular filtration rate was evaluated 24 hours before and after the procedure. After procedure, blood urea nitrogen in all the three groups decreased; Scr of S-C group and I-A group increased significantly, while estimated glomerular filtration decreased in the S-C group (*P* < 0.05); serum uric acid in S-C group and I-C group decreased significantly (*P* < 0.05). Combination treatment of atorvastatin and probucol before intervention could reduce perioperative serum uric acid level; meanwhile, the intensive combined treatment can improve the contrast induced acute kidney injury. The result was the same for hypertensive patients.

## 1. Introduction

Contrast induced acute kidney injury (CI-AKI) is reported to be the third leading cause of in-hospital acute renal failure [1–3]. Serum uric acid (SUA) could stimulate renin-angiotensin-aldosterone system [4], which plays an important role in the pathogenesis of CI-AKI. Hyperuricemia has ever been considered as its risk factor [5]. Recently, researches have shown that atorvastatin and probucol could improve CI-AKI separately [6–8]. However, little is known about the effect of pretreatment with atorvastatin combined with probucol on SUA level in patients who experience coronary angiography (CAG) or percutaneous coronary intervention (PCI). Therefore, in this study, we investigated the effects of different doses of atorvastatin combined with fixed dose of probucol on the level of SUA and its role in improving CI-AKI.

## 2. Material and Methods

### 2.1. Patients

From May 2010 till December 2010, two hundred and eight patients who underwent CAG or PCI for coronary heart disease in Tianjin Chest Hospital of China were evaluated consecutively. Blood urea nitrogen (BUN), serum creatinine (Scr), and serum uric acid (SUA) were measured before operation. Estimated glomerular filtration rate (eGFR) was estimated using the modified diet in renal disease (MDRD) study formula. The study population was randomly assigned into three groups: standard combined treatment group (S-C group, *n* = 55), intensive combined treatment group (I-C group, *n* = 79), and intensive atorvastatin therapy group (I-A group, *n* = 74).

### 2.2. Diagnosis Criteria

Diagnosis criteria of coronary heart disease in accordance with the 2007 Chinese chronic stable angina pectoris diagnosis and treatment guidelines [9], 2007 Chinese unstable angina and non-ST segment elevation myocardial infarction diagnosis and treatment guidelines [10], and universal definition of myocardial infarction in 2008 [11]. CI-AKI is defined as a postprocedural increase in serum creatinine of ≥44.2 *μ*mol/L (0.5 mg/dL) or >25% from baseline [1, 12, 13].

### 2.3. Study Design

The hypotonic nonionic contrast media iopamidol injection (ShangHai boLecco, Xinyi Pharmaceutical Co., Ltd.) was used in patients. Baseline clinical and procedural characteristics were collected before procedure including the age, gender, body mass index, history of hypertension, and diabetes; the biochemical indicators such as lipid profile and blood glucose were measured, and all patients' followup BUN, Scr, and SUA were tested 24 hours before and 24 hours after the procedure. All patients received the treatment of aspirin, clopidogrel, heparin, nitrate, angiotensin converting enzyme inhibitor (ACEI), and *β* blockers after admission. All eligible patients started to receive atorvastatin or atorvastatin with probucol 1-2 days before operation. The S-C group took atorvastatin 20 mg qn and probucol 0.25 g tid, with no loading dose intake before procedure; the I-C group took atorvastatin 40 mg qn and probucol 0.25 g tid, with a loading dose of atorvastatin 40 mg and probucol 0.5 g 2 hours before the operation; the I-A group took atorvastatin 40 mg qn, with a loading dose of atorvastatin 40 mg 2 hours before the operation. After the procedure, they continued to take the same doses of atorvastatin and probucol. All patients received hydration therapy with normal saline at a rate of 1 mL·kg^−1^·h^−1^ for 6 hours before and 6 hours after procedure. The study was approved by the medical ethics committee of Tianjin Chest Hospital of China. All patients provided written informed consent, and the study was carried out in accordance with the Declaration of Helsinki.

### 2.4. Exclusion Criteria

Patients who underwent PCI for acute myocardial infarction were excluded. The other exclusion criteria included alanine transaminase ≥80 U/L, serum creatinine > 264 *μ*mol/L, cancer patients, blood diseases or autoimmune diseases, cardiogenic shock, and left ventricular ejection fraction ≤30%, gout, history of hypersensitivity to contrast media, atorvastatin or probucol, prolonged QT interval (corrected QT interval > 0.44 s), previous contrast media exposure within 7 days of study entry, pregnancy, or lactation. Also, patients who had used diuretics during hospitalization or used probenecid, benzbromarone, or allopurinol which affect SUA were excluded. Furthermore, patients who had used statins or probucol within 30 days or had used *N*-acetylcysteine or nonsteroidal anti-inflammatory drugs were all excluded.

### 2.5. Statistical Analysis

Statistical analysis was performed using the SPSS (version 17.0). Continuous data were reported as mean ± standard deviation. Main statistical indicators were tested for normality and homogeneity of variance. The *t*-test of paired samples was used to compare differences within groups following an intervention. Analysis of variance was used to compare differences among various groups; in addition, the method of Students-Newman-Keuls (SNK) was used to reveal the differences among groups. Categorical data were presented as percentages. Chi-square test was performed for the comparison of categorical variables as priority. A *P* value of <0.05 (2-sided) was considered to reflect statistical significance.

## 3. Results

### 3.1. Patients and Baseline Characteristics

The baseline data are shown in Table 1.

### 3.2. Markers of Renal Function (the Change of Scr, BUN, and eGFR between Three Groups)

Baseline renal function including BUN, Scr, SUA, and eGFR of the three groups were similar (*P* > 0.05). After operation, BUN in all groups decreased; Scr in S-C group and I-A group increased significantly, while only the S-C group's eGFR decreased (*P* < 0.05); there were no significant differences in Scr and eGFR of I-C group after the procedure (*P* > 0.05). SUA in S-C group and I-C group decreased significantly after operation (*P* < 0.05), while no significant difference was observed in I-A group (*P* > 0.05). The absolute change of UA (ΔUA) in S-C group and in I-C group was significantly higher than I-A group (both *P* < 0.05). There were two patients who suffered from CIAKI; one was in S-C group and the other was in I-C group (Table 2).

### 3.3. Postoperation Changes of Renal Parameters in the Hypertension Subgroup

According to ESH and ESC Guidelines of Hypertension [14] in 2007, we selected 140 hypertensive patients to analyze changes of renal parameters before and after operation in the subgroup. eGFR in S-C group and I-A group decreased significantly (*P* < 0.05), while SUA showed no significant difference in the two groups; in I-C group, BUN and SUA decreased markedly (*P* < 0.05), while Scr and eGFR showed no significant changes (Table 3).

## 4. Discussion

Intravascular administration of iodinated contrast media gives rise to a potential danger of renal hemodynamic instability as a cause of renal ischemia, hypoxia, and oxidative inflammatory response, thus bringing about impaired renal function [15]. Retrospective studies have reported CI-AKI incidence ranging from 1.5% to 10% of general population, while the incidence increased to 30–50% with high risk factors such as chronic renal insufficiency, diabetes, and hypertension [16–18]. In our study, two patients suffered CI-AKI, occurred in 0.96% (2 of 208) of the overall studied population. The lower incidence was probably for the reason that we only examined serum creatinine level once after operation. Uric acid is greatly important in the pathogenesis of CI-AKI. It has been suggested that tubular obstruction by uric acid plays a role in the pathogenesis of CI-AKI [19, 20]. Furthermore, hyperuricemia is accompanied by enhanced synthesis of reactive oxygen species, an activated renin-angiotensin-aldosterone system, increased endothelin-1, and inhibited nitric oxide system [21, 22], which results in strong constriction of blood vessels further reducing the blood flow to the renal medulla. In addition, xanthine oxidase is an important source of superoxide free radicals, which plays a crucial role to increase the production and activity of uric acid. Elevated uric acid, accompanied with inflammatory reaction and oxidative stress, is involved in the pathogenesis of CI-AKI [23]. Toprak et al. [5] reported that in a total of 266 patients with Scr ≥1.2 mg/dL who underwent coronary angiography, CI-AKI occurred at 15.1% in the hyperuricemic group and 2.9% in the normouricemic group (*P* < 0.001). Recent epidemiologic and experimental evidence suggests the role of SUA is not only an independent cardiovascular risk factor [24] but also a causal risk factor for the development and progression of renal disease. Elevated levels of uric acid independently increase the risk of new-onset kidney disease [25]. Hyperuricemia is associated with the disease occurrence of vital organs such as blood vessels, heart, and kidneys. Therefore, reducing uric acid is expected to be a new way to prevent cardiovascular disease. Epidemiologic studies suggest that high level of SUA is an independent risk factor of hypertension [26]. Hypertensive nephropathy may increase the level of SUA. Hypertension complicated with hyperuricemia could affect each other, resulting in renal function damage.

The prophylactic effects of statin treatment on the development of CI-AKI are still controversial. The recent systemic review and meta-analysis have not provided conclusive result [27]. Under the fixed-effects model, a nonsignificant protective trend toward decreased incidence of CIN with periprocedural short-term high-dose statin treatment was seen (RR: 0.70; 95% CI: 0.48–1.02). Nevertheless, there is a great body of studies that have proved that statin treatment can effectively prevent CI-AKI [28, 29]. In Su et al.'s study [30], multivariate logistic regression analysis showed that pretreatment with high dose atorvastatin was a protective factor for post-CI-AKI (20 mg atorvastatin: *P* = 0.001; 40 mg atorvastatin: *P* = 0.001). The mechanism may be that atorvastatin can improve eGFR, remove free radicals, inhibit the inflammatory response [31, 32], and reduce uric acid level by increasing its excretion [33]. Probucol plays a significant role in improving the CI-AKI owing to clearing oxygen free radicals, against oxidative stress, thus improving endothelial function [8]. Currently, there is a lack of large-scale researches about the effect on SUA of atorvastatin combined with probucol intake during perioperative intervention. Our small sample research suggested that a certain dose of atorvastatin and probucol intake in short-term preoperatively can decrease the perioperative SUA level significantly. The BUN in I-C group decreased, while there was no significant difference in Scr and eGFR. The SUA in I-A group did not decrease, while the Scr increased without eGFR decreasing. The analysis of hypertensive subgroup suggested that intensive atorvastatin and probucol combination did not change postoperative Scr and eGFR significantly, but could reduce perioperative SUA level significantly. According to our study, combination treatment of atorvastatin and probucol before intervention could reduce perioperative SUA level; further the intensive combined treatment can improve CI-AKI. For hypertensive patients, combination and intensive treatment could reduce SUA level and improve the CI-AKI. Thus, patients with coronary heart disease or hypertension who are undergoing the intervention may benefit from the combined medication by reducing the uric acid level and improving CI-AKI.

The major limitations of the present study were the short duration of observation and having a small sample. Also, this was a single-center observational clinical study. Large sample and multicenter trials ought to be designed to verify the conclusion.

## Figures and Tables

**Table 1:** Baseline clinical, biochemical and procedural characteristics of the patients.

Variable	S-C group (*n* = 55)	I-C group (*n* = 79)	I-A group (*n* = 74)	*P* value
Age (year)	62.33 ± 9.49	60.65 ± 10.11	61.00 ± 10.23	0.616^a^
Female gender	25 (45.45%)	33 (41.72%)	27 (36.49%)	0.579^b^
Body mass index (kg/m^2^)	27.24 ± 10.23	25.96 ± 3.51	28.35 ± 17.26	0.454^a^
Current smoking (*n*)	19/34.55	33/41.77	36/48.65	0.274^b^
Total cholesterol (mmol/L)	4.81 ± 1.21	4.91 ± 1.59	4.68 ± 1.27	0.592^a^
Triglyceride (mmol/L)	1.83 ± 0.96	1.71 ± 0.78	1.67 ± 0.93	0.553^a^
Low-density lipoprotein (mmol/L)	2.74 ± 0.92	2.98 ± 1.32	2.72 ± 1.02	0.291^a^
Blood glucose (mmol/L)	5.66 ± 1.56	5.62 ± 1.71	5.65 ± 2.00	0.993^a^
Red blood cell count (10^12^/L)	4.50 ± 0.76	4.53 ± 0.41	4.35 ± 0.46	0.116^ a^
White blood cell count (10^9^/L)	6.91 ± 1.53	7.29 ± 1.87	7.73 ± 1.92	0.211^a^
Diabetes mellitus (*n*)	11 (20.00%)	23 (29.11%)	16 (21.62%)	0.398^b^
Volume of contrast agent (mL)	187.96 ± 103.22	185.53 ± 142.32	185.71 ± 123.69	0.993^ a^
Hypertension (*n*)	38 (69.09%)	53 (67.09%)	49 (66.22%)	0.548^b^

Values are expressed as means ± SD or number (%) of patients.^ a^By Student's *t* test; ^b^By *χ* ^2^ test.

**Table 2:** Comparison of serum BUN, Scr, SUA and eGFR levels at baseline and 24 hours after Intervention.

Variable	S-C group	I-C group	I-A group	*P*′ value
BUN (mmol/L)				
Baseline	5.66 ± 1.48	5.58 ± 1.29	5.27 ± 1.27	0.203
Post procedural	4.93 ± 2.08	4.63 ± 1.17	4.96 ± 1.25	—
Absolute change ΔBUN	−0.72 ± 1.77	−0.95 ± 1.24	−0.31 ± 1.32	0.022
*P* value	0.004	0.000	0.046	—
Scr (umol/L)				
Baseline	82.45 ± 15.48	81.77 ± 18.37	85.83 ± 15.45	0.287
Post procedural	88.32 ± 24.68	82.67 ± 16.88	88.66 ± 16.87	—
Absolute change ΔScr	5.87 ± 19.36	0.89 ± 12.96	2.84 ± 9.47	0.129
*P* value	0.029	0.542	0.012	—
SUA (mmol/L)				
Baseline	322.36 ± 77.57	310.45 ± 69.59	329.77 ± 81.88	0.289
Post procedural	299.32 ± 72.16	281.20 ± 62.80	328.11 ± 90.79	—
Absolute change ΔSUA	−23.04 ± 64.80	−29.25 ± 43.07	−1.66 ± 83.03	0.028
*P* value	0.011	0.000	0.864	—
eGFR (mL/min)				
Baseline	79.12 ± 13.49	81.64 ± 15.70	78.67 ± 16.19	0.444
Post procedural	75.33 ± 15.70	80.47 ± 15.33	76.28 ± 15.72	

*P* value was the comparison of the group between Baseline and Post procedural. *P*′ value was the comparison among groups.

**Table 3:** Post-operation changes of renal parameters in the hypertensive subgroup.

Variable	S-C group (*n* = 38)	I-C group (*n* = 53)	I-A group (*n* = 49)	*P*′ value
BUN (mmol/L)				
Baseline	5.61 ± 1.29	5.61 ± 1.32	5.37 ± 1.21	0.556
Post procedural	4.86 ± 1.85	4.62 ± 0.99	5.12 ± 1.35	—
Absolute change ΔBUN	−0.74 ± 1.85	−0.99 ± 1.17	−0.25 ± 1.23	0.027
*P* value	0.017	0.000	0.161	—
Scr (*μ*mol/L)				
Baseline	81.85 ± 15.80	78.60 ± 15.34	85.37 ± 16.26	0.100
Post procedural	88.80 ± 25.33	80.73 ± 17.04	89.79 ± 18.09	—
Absolute change ΔScr	6.94 ± 21.79	2.13 ± 12.08	4.42 ± 9.20	0.300
*P* value	0.054	0.205	0.002	—
SUA (mmol/L)				
Baseline	328.93 ± 78.64	314.88 ± 70.97	331.88 ± 86.54	0.512
Post procedural	308.28 ± 73.35	285.05 ± 66.12	325.60 ± 72.63	—
Absolute change ΔSUA	−20.65 ± 65.25	−29.82 ± 45.78	−6.28 ± 68.88	0.143
*P* value	0.055	0.000	0.527	—
eGFR (mL/min)				
Baseline	79.68 ± 13.14	83.29 ± 15.29	77.87 ± 14.91	0.165
Post procedural	74.67 ± 14.36	81.44 ± 15.70	73.75 ± 14.80	
Absolute change ΔScr	−5.01 ± 11.07	−1.85 ± 10.47	−4.11 ± 9.17	0.304
*P* value	0.008	0.203	0.003	

*P* value was the comparison of the group between Baseline and Post procedural. *P*′ value was the comparison among groups.

## References

[1] McCullough PA, Soman SS (2005). Contrast-induced nephropathy. *Critical Care Clinics*.

[2] Persson PB, Hansell P, Liss P (2005). Pathophysiology of contrast medium-induced nephropathy. *Kidney International*.

[3] Detrenis S, Meschi M, Musini S, Savazzi G (2005). Lights and shadows on the pathogenesis of contrast-induced nephropathy: state of the art. *Nephrology Dialysis Transplantation*.

[4] Perlstein TS, Gumieniak O, Hopkins PN (2004). Uric acid and the state of the intrarenal renin-angiotensin system in humans. *Kidney International*.

[5] Toprak O, Cirit M, Esi E, Postaci N, Yesil M, Bayata S (2006). Hyperuricemia as a risk factor for contrast-induced nephropathy in patients with chronic kidney disease. *Catheterization and Cardiovascular Interventions*.

[6] Özhan H, Erden I, Ordu S (2010). Efficacy of short-term high-dose atorvastatin for prevention of contrast-induced nephropathy in patients undergoing coronary angiography. *Angiology*.

[7] Shepherd J, Kastelein JJ, Bittner V (2007). Effects of intensive lipid lowering with atorvastatinon renal function: the treating to new targets study. *Clinical Journal of the American Society of Nephrology*.

[8] Yin L, Li G-P, Liu T (2009). Role of probucol in preventing contrast induced acute kidney injury after coronary interventional procedure: a randomized trial. *Chinese Journal of Cardiovascular Diseases*.

[9] Cardiovascular branch of Chinese Medical Association (2007). chronic stable angina pectoris diagnosis and treatment guidelines. *Chinese Journal of Cardiology*.

[10] Cardiovascular branch of Chinese Medical Association (2007). Unstable angina and non ST segment elevation myocardial infarction diagnosis and treatment guidelines. *Chinese Journal of Cardiology*.

[11] Cardiovascular branch of Chinese Medical Association (2008). Recommendation the use of universal definition of myocardial infarction in China. *Chinese Journal of Cardiology*.

[12] Mehran R, Kahn J (2007). Contrast-induced nephropathy remains a serious complication of PCI. *Journal of Interventional Cardiology*.

[13] McCullough PA (2003). Beyond serum creatinine: defining the patient with renal insufficiency and why?. *Reviews in Cardiovascular Medicine*.

[14] The task force for he management of arterial hypertension of the European Society of hypertension (ESH) and of the European Society of Cardiology (ESC) (2007). 2007 Guidelines for the management of arterial hypertension. *Journal of Hypertension*.

[15] Sterling KA, Tehrani T, Rudnick MR (2008). Clinical significance and preventive strategies for contrast-induced nephropathy. *Current Opinion in Nephrology and Hypertension*.

[16] Amini M, Salarifar M, Amirbaigloo A, Masoudkabir F, Esfahani F (2009). N-acetylcysteine does not prevent contrast-induced nephropathy after cardiac catheterization in patients with diabetes mellitus and chronic kidney disease: a randomized clinical trial. *Trials*.

[17] Murphy SW, Barrett BJ, Parfrey PS (2000). Contrast nephropathy. *Journal of the American Society of Nephrology*.

[18] Gleeson TG, Bulugahapitiya S (2004). Contrast-induced nephropathy. *The American Journal of Roentgenology*.

[19] Postlethwaite AE, Kelley WN (1971). Uricosuric effect of radiocontrast agents: a study in man of four commonly used preparations. *Annals of Internal Medicine*.

[20] Mudge GH (1971). Uricosuric action of cholecystographic agents: a possible factor in nephrotoxicity. *The New England Journal of Medicine*.

[21] Kang D-H, Yu ES, Park J-E (2005). Uric acid induced C-reactive protein expression via upregulation of angiotensin type 1 receptors in vascular endothelial cells and smooth muscle cells. *Journal of the American Society of Nephrology*.

[22] Johnson RJ, Kang D-H, Feig D (2003). Is there a pathogenetic role for uric acid in hypertension and cardiovascular and renal disease?. *Hypertension*.

[23] Ruggiero C, Cherubini A, Ble A (2006). Uric acid and inflammatory markers. *European Heart Journal*.

[24] Bos MJ, Koudstaal PJ, Hofman A, Witteman JCM, Breteler MMB (2006). Uric acid is a risk factor for myocardial infarction and stroke: the Rotterdam Study. *Stroke*.

[25] Obermayr RP, Temml C, Gutjahr G, Knechtelsdorfer M, Oberbauer R, Klauser-Braun R (2008). Elevated uric acid increases the risk for kidney disease. *Journal of the American Society of Nephrology*.

[26] Sundström J, Sullivan L, D’Agostino RB, Levy D, Kannel WB, Vasan RS (2005). Relations of serum uric acid to longitudinal blood pressure tracking and hypertension incidence. *Hypertension*.

[27] Zhang T, Shen L-H, Hu L-H, He B (2011). Statins for the prevention of contrast-induced nephropathy: a systematic review and meta-analysis. *The American Journal of Nephrology*.

[28] Xinwei J, Xianghua F, Jing Z (2009). Comparison of usefulness of simvastatin 20 mg versus 80 mg in preventing contrast-induced nephropathy in patients with acute coronary syndrome undergoing percutaneous coronary intervention. *The American Journal of Cardiology*.

[29] Zhou X, Jin Y-Z, Wang Q, Min R, Zhang X-Y (2009). Efficacy of high dose atorvastatin on preventing contrast induced nephropathy in patients underwent coronary angiography. *Chinese Journal of Cardiovascular Diseases*.

[30] Su JZ, Xue Y, Cai WQ (2011). Association between high sensitivity C-reactive protein and contrast induced acute kidney injury in patients with acute coronary syndrome undergoing percutaneous coronary intervention: impact of atorvastatin. *Chinese Journal of Cardiovascular Diseases*.

[31] Khanal S, Attallah N, Smith DE (2005). Statin therapy reduces contrast-induced nephropathy: an analysis of contemporary percutaneous interventions. *The American Journal of Medicine*.

[32] Patti G, Nusca A, Chello M (2008). Usefulness of statin pretreatment to prevent contrastinduced nephropathy and to improve long-term outcomes in patients undergoing percutaneous coronary intervention. *The American Journal of Cardiology*.

[33] Milionis HJ, Kakafika AI, Tsouli SG (2004). Effects of statin treatment on uric acid homeostasis in patients with primary hyperlipidemia. *The American Heart Journal*.

